# Place and cause of death among people with chronic neurological diseases in Munich: a population-based study using death certificates

**DOI:** 10.1186/s42466-026-00536-x

**Published:** 2026-10-06

**Authors:** Sarah Kristina Bublitz, Claudia Bausewein, Veronika Detter, Berend Feddersen, Matthias Graw, André Klima, Patricia Klotz, Stefan Lorenzl, Sabine Gleich

**Affiliations:** 1 Institute of Palliative Care, Paracelsus Medical Private University, Salzburg, Austria; 2 Agatharied Hospital, Hausham, Germany; 3 Department of Palliative Medicine, LMU University Hospital, LMU Medizin, Ludwig- Maximilians-Universität München, Munich, Germany; 4 Department of Forensic Medicine, LMU University Hospital, LMU Medizin, Ludwig- Maximilians-Universität München, Munich, Germany; 5 Department of Public Health, Munich, Germany

**Keywords:** Palliative care, Neuropalliative care, Amyotrophic lateral sclerosis, Parkinson’s disease, Goal-concordant care, Death certificates

## Abstract

**Introduction:**

Access to palliative care is often delayed and fragmented for patients with chronic neurological diseases. This study aimed to characterize the place and cause of death among individuals with chronic neurological diseases to help identify potential gaps in palliative care provision.

**Method:**

In this cross-sectional study, all death certificates from the city of Munich, Germany (2020–2023) were reviewed. Cases were included if malignant brain tumours, amyotrophic lateral sclerosis (ALS) or other motor neuron diseases, Parkinson’s disease (PD), multiple sclerosis (MS) or other neurodegenerative or neuromuscular diseases were recorded as the cause of death or as a comorbidity.

**Results:**

Of 60,357 deaths, 2,536 individuals (4.2%) had at least one chronic neurological disease. The most common place of death was the hospital (43.8%), followed by nursing homes (25.9%) and the private home (25.0%), with variations among disease subgroups. Overall, the neurological disease was recorded as the immediate or underlying cause of death in 57.3% of cases, while in the remaining cases it was documented as a comorbidity, reflecting its presence without formal causal attribution to death.

**Discussion:**

This population-level analysis provides insight into the place and circumstances of death for a large cohort of patients with chronic neurological diseases in a major German metropolitan area and highlights substantial heterogeneity in end-of-life trajectories. The findings indicate that mortality statistics based on death certificates reflect structural limitations in cause-of-death attribution. Nevertheless, place of death remains a robust indicator, showing high hospital utilization and limited hospice use across most diagnostic groups, underscoring potential gaps in goal-concordant care.

## Introduction

Preferred place of death can be considered a key indicator of how well palliative care systems enable goal-concordant and patient-centred end-of-life (EOL) care. Although a majority of people express a preference to die at home [1], a substantial proportion still die in acute hospitals [2], suggesting that preferences often remain unmet and that barriers to community-based palliative care persist. Cancer patients often receive earlier and more structured palliative care than non-cancer patients [3], which is associated with higher rates of home or hospice death and fewer hospitalizations near the EOL [4]. In contrast, individuals with non-oncological chronic progressive diseases frequently experience delayed or fragmented access to palliative care services, as well as prognostic uncertainty, practical obstacles and high caregiver burden. These factors increase hospital deaths and reduce the likelihood of dying in a preferred setting. Among non-oncological diseases, neurological conditions pose particular challenges for palliative care provision. Patients with neurological diseases face disease-specific barriers, including rapid or unpredictable progression, communication loss, cognitive decline, dependency on technical and family support and challenges faced by healthcare professionals in recognizing the end-of-life phase. Their palliative care needs can begin at the time of diagnosis and fluctuate in a non-linear way over the course of the disease [5]. Recent findings from specialized ALS centres in Germany further indicate that clinical challenges are compounded by structural barriers: collaboration with specialized palliative care (SPC) is often informal and not standardized, and predefined criteria for the integration of SPC in ALS care are lacking [6]. Patients with neurodegenerative diseases are more likely to be submitted to the emergency department and are less likely to use hospice service at the end of life than persons with cancer [7].

While some German national data on place of death among people with neurological diseases exist [8], regional patterns reflecting local health system capacity and infrastructure are likely to be a major influence factor. Examining place and cause of death among major neurological diseases at a regional level can illuminate diagnosis-specific barriers and inform the development of equitable, needs-based palliative care models. Therefore, this study aimed to characterize the mortality profile of individuals with chronic neurological diseases in Munich, Germany, representing a large, well-resourced urban healthcare setting. The core objectives were to describe the demographic characteristics, place of death and causes of death. By detailing these distributions, we sought to determine how local health-system factors can potentially shape EOL trajectories and to identify specific areas where the delivery of palliative care for people with neurological conditions may be optimized.

## Methods

### Design

In this cross-sectional study, death certificates of all people who died in the city of Munich, Germany, in the four-year-period of 2020 until 2023 were analysed.

Data were handled in compliance with all medical confidentiality requirements in accordance with the recommendations of the German Medical Association and Sect.  203 of the German Criminal Code (breach of private secrets). The study received approval from the local Research Ethics Committee at the Medical Faculty of Ludwig- Maximilians- University in Munich (project number 24–0969). The STROBE reporting guidelines were followed.

### Setting and participants

All death certificates of individuals who died in the municipality of Munich, Germany, between January 1, 2020, and December 31, 2023, and which are archived in paper form by the Munich Health Department, were reviewed. In Bavarian death certificates, the physician who performs the external post-mortem examination inserts information on the place of death and classifies the condition as (immediate or underlying) cause of death, or as a contributing comorbidity (please see Table 1 for the essential contents of Bavarian death certificates used for this analysis).

**Table 1:** Essential contents of Bavarian death certificates used for data extraction in this study

Contents of Bavarian Death Certificates used for data extraction	
Personal data	• Date of birth, sex • Date and time of death (If time of death is unknown: time of discovery; last seen alive) • Place of death (private address, nursing home, hospice, disability support facility, official detention, hospital) • Type of death (natural, undetermined, non-natural)
Reanimation	Yes/no
Medical information: most likely cause of death / clinical findings	I. a) Disease or condition directly leading to death b) Due to (Antecedent causes – conditions, if any, leading to the immediate cause of death) c) Underlying cause/disease II. Other significant conditions (contributing to death)

Inclusion criteria were the indication of malignant brain tumours, Amyotrophic Lateral Sclerosis (ALS) or other motor neuron diseases, Parkinson’s disease (PD), multiple sclerosis (MS) or other neuromuscular or neurodegenerative diseases under the topics I.a or I.c (immediate or underlying cause of death) or II. (comorbidity) in the confidential section of the death certificate. Dementias were explicitly excluded from the analysis due to methodological constraints because of high variance in documentation.

### Data entry and analysis

A study-specific data extraction matrix was developed and piloted using death certificates from a ten-day period (367 cases; 2.7% of the total study period). After minor adjustments, data were entered by hand into Microsoft Excel (Microsoft Office 2011) after carefully reviewing each paper-based death certificate. All data were subsequently anonymized and analysed descriptively using SPSS (IBM, Armonk, NY, USA, version 26) and R (R Foundation for Statistical Computing, Vienna, Austria, https://www.R-project.org, version 4.3.1). For testing mean differences in age between groups, ANOVA was used. For each group, the number of observations was well above 30. Difference between groups for categorical variables were tested using Chi²-tests. When Chi²-approximation was not considered valid, p-values were estimated using Monte Carlo simulation (100.000 replication).

## Results

### Characteristics of the total cohort

During the study period, 60,357 deaths were recorded in the City of Munich. According to the publicly available cause-of-death statistics from the German Federal Statistical Office (Todesursachenstatistik des Deutschen Statistischen Bundesamtes, www.gbe-bund.de), the total number of deaths in Germany during this period was 4,103,806. Hence, our data correspond to 1.5% of all deaths in the Federal Republic of Germany. Of the 60,357 cases in Munich, 2,536 people (4.2%) met the inclusion criteria by having at least one chronic neurological disease.

Table 2 shows demographic characteristics of the total study cohort. In the vast majority, the manner of death was assessed as natural by the certifying physician. In cases of death occurring in hospital, the death certification was performed by a hospital physician (43.8%), but death certificates do not provide information on the physician’s specialty or level of qualification. For deaths occurring in nursing homes, private residences or hospices, the post-mortem examination may be performed by general practitioners or other community-based physicians (47.9% of the total cohort) or physicians working in specialized palliative home care (SAPV) teams. SAPV physicians only perform the examination if the patient has been under their team’s care at the time of death. In the total cohort, SAPV physicians account for 7.2% of certifying physicians. Autopsies were rarely performed and, when conducted, predominantly court-ordered due to suspected external causes of death (non-natural causes, such as falls or bolus aspiration).

In the total cohort of 2,536 people with a chronic neurological disease, the most common place of death was the hospital (43.8%), with equal proportions of death occurring on intensive or intermediate care units and on palliative care units, followed by the private home or nursing home. Only a small minority of 4.8% died in hospice, and only few died in a residential intensive care facility or other places.

**Table 2:** Demographic data and places of death in total cohort

Demographic data of the total cohort (*n* = 2,536)	
Age (mean/median/range) in years	76.9/80/0-101
Male	1,550 (61.1%)
Female	986 (38.9%)
Nursing home resident	828 (32.6%)
Death certificate by physician from SAPV team	183 (7.2%)
Autopsy performed Among these Forensic autopsies	61 (2,4%) 51 (83,6%)
**Place of death**,** all cases**	
Hospital	1,112 (43.8%)
Intensive or intermediate care unit	247/1,112 (22.3%)
Palliative care unit	248/1,112 (22.3%)
Private home	635 (25.0%)
Nursing home	657 (25.9%)
Hospice	123 (4.8%)
Residential intensive care facility	3 (0.1%)
Other places	6 (0.2%)

Respiratory diseases were the most common immediate cause of death in about every fourth patient of the total cohort and in more than a third, the neurological disease was regarded as the underlying cause of death. For details on the sequencing cause of death, please refer to Table 3. Although terminal conditions or terminal physiological mechanisms, such as “cardiorespiratory failure”, are not permitted as underlying causes of death under German death certification regulations, they are nevertheless frequently recorded in practice, likely reflecting diagnostic uncertainty regarding the causal chain leading to death.

As the study period coincided with the COVID-19 pandemic, cases in which COVID-19 was recorded as the immediate or underlying cause of death were analyzed in the total cohort with neurological diseases separately. Overall, 5% of deaths in the study population were COVID-19-associated. Of the 25 cases with COVID-19 as the immediate cause of death, 14 were nursing home residents; 9 died in the nursing home, 14 in hospital, and 2 at a private residence. Of the 73 cases with COVID-19 as the underlying cause of death, 26 were nursing home residents; 11 died in the nursing home, 58 in hospital, 3 at a private residence, and 1 in a hospice.

**Table 3:** Immediate or underlying causes of death in total cohort

All Cases with chronic neurological diseases	*n* = 2,536 (100%)
*Immediate cause of death (according to ICD-10)*	
A00-B99 Infectious and parasitic diseases *COVID-19*	234 (9.2%) *25*
C00-C97 Neoplasms *malignant brain tumours (glioblastoma*,* astrocytoma)*	154 (6.1%) *125 (4.9%)*
D50-D89 Diseases of the blood and blood-forming organs	9 (0.3%)
E00-E90 Endocrine, nutritional and metabolic diseases	24 (0.9%)
F00-F99 Mental, Behavioral and Neurodevelopmental disorders	12 (0.5%)
G00-H95 Diseases of the nervous system *ALS/Motoneuron diseases* *M. Parkinson and atypical Parkinson’s syndromes* *Multiple Sklerose* *Other neuromuscular diseases (muscular dystrophies*,* myopathies*,* myotonias*,* neuropathies)* *Other neurodegenerative diseases except dementias (hereditary ataxias*,* M. Huntington*,* Creutzfeld-Jakob-disease*,* etc.)* *other*	158 (6.3%) *34 (1.3%)* *32 (1.3%)* *9 (0.4%)* *3 (0.1%)* *5 (0.2%)* *75 (3.0%)*
I00-I99 Diseases of the circulatory system	269 (10.6%)
J00-J99 Diseases of the respiratory system *Pneumonia (bacterial*,* viral)* *Aspiration pneumonia*	692 (27.3%) *257 (10.1%)* *351 (13.8%)*
K00-K93 Diseases of the digestive system	65 (2.6%)
M00-M99 Diseases of the musculoskeletal system and connective tissue	-
N00-N99 Diseases of the genitourinary system	133 (5.2%)
R95-R99 Symptoms, signs and abnormal clinical and laboratory findings, not elsewhere classified	140 (5.5%)
T17 Foreign body in respiratory tract	18 (0.7%)
V01-Y98 External causes of morbidity and mortality *Falls* *Suicide* *Assisted suicide*	33 (1.3%) *6 (0.2%)* *7 (0.3%)* *16 (0.6%)*
Terminal physiological mechanism*	570 (22.5%)
No information	25 (1.0%)
*Underlying cause of death (according to ICD-10)*	
A00-B99 Infectious and parasitic diseases *COVID-19*	99 (3.9%) *73*
C00-C97 Neoplasms *malignant brain tumours (glioblastoma*,* astrocytoma)*	396 (15.6%) *319 (12.6%)*
D50-D89 Diseases of the blood and blood-forming organs	3 (0.1%)
E00-E90 Endocrine, nutritional and metabolic diseases	18 (0.7%)
F00-F99 Mental, Behavioural and Neurodevelopmental disorders	57 (2.2%)
G00-H95 Diseases of the nervous system *ALS/Motoneuron diseases* *M. Parkinson and atypical Parkinson’s syndromes* *Multiple Sclerosis* *Other neuromuscular diseases (muscular dystrophies*,* myopathies*,* myotonias*,* neuropathies)* *Other neurodegenerative diseases except dementias (hereditary ataxias*,* M. Huntington*,* Creutzfeld-Jakob-disease*,* etc.)* *other*	953 (37.6%) *123 (4.8%)* *684 (27.0%)* *69 (2.7%)* *43 (1.7%)* *22 (0.9%)* *11 (0.4%)*
I00-I99 Diseases of the circulatory system	267 (10.5%)
J00-J99 Diseases of the respiratory system *Pneumonia (bacterial*,* viral)* *Aspiration pneumonia*	84 (3.3%) - *23 (0.9%)*
K00-K93 Diseases of the digestive system	60 (2.4%)
M00-M99 Diseases of the musculoskeletal system and connective tissue	10 (0.4%)
N00-N99 Diseases of the genitourinary system	60 (2.4%)
R95-R99 Symptoms, signs and abnormal clinical and laboratory findings, not elsewhere classified	110 (4.3%)
T17 Foreign body in respiratory tract	3 (0.1%)
V01-Y98 External causes of morbidity and mortality *Falls* *Other accidents* *Suicide* *Assisted suicide*	34 (1.3%) *24 (0.9%)* *2 (0.1%)* *-* *3 (0.1%)*
Terminal physiological mechanism*	12 (0.5%)
No information	370 (14.7%)

*What is referred to as “terminal physiological mechanism” reflects categories such as cardiorespiratory failure

### Neurological diseases as the immediate or underlying cause of death

In 57.3% (1,454/2,536), the chronic neurological disease was regarded as the immediate or underlying cause of death, i.e. as causally leading to death. Parkinson’s disease was the most common neurological disease as causative for death in this cohort, followed by brain tumours, ALS and MS (Table 4).

**Table 4:** Analysis of demographic data, inspection of corpse and autopsies of four subgroups

	Brain tumour *n* = 434	ALS *n* = 152	Parkinson’s disease *n* = 716	MS *n* = 78	*p*-value
Age (mean/median/range) in years	64.1/68/0–96	70.8/71/38–93	82.0/82/52–101	69.1/70/43–94	< 0.001*
Male	272 (62.7%)	82 (53.9%)	477 (66.6%)	31 (39.7%)	< 0.001°
Nursing home resident	47 (10.8%)	19 (12.5%)	298 (41.6%)	29 (37.2%)	< 0.001°
Certifying physician from SAPV team	58 (13.4%)	26 (17.1%)	54 (7.5%)	2 (2.6%)	
Autopsy performed (%) [forensic autopsies]	3 (0.7%) [2]	0	12 (1.7%) [11]	1 (1.3%) [1]	

For this analysis, only cases were analysed, in which one of the four diseases (brain tumour, ALS, Parkinson’s disease or Multiple Sclerosis) was regarded as causative for death

SAPV: specialized palliative home care teams (“Spezialisierte Ambulante Palliativversorgung”)

* ANOVA Test; °Chi^2^-Test

**Fig. 1:**
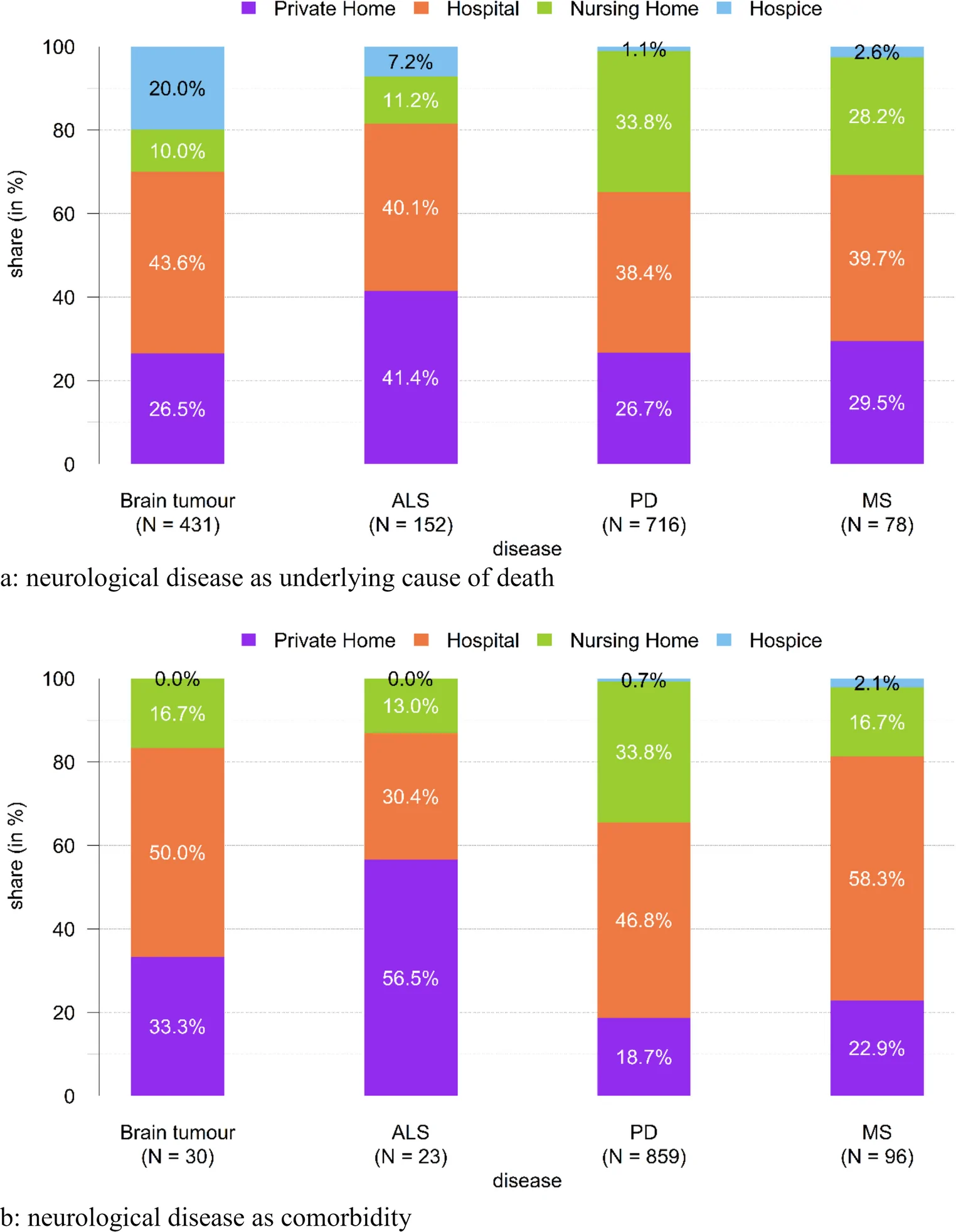
Distribution of place of death (%) among individuals with brain tumour, Amyotrophic Lateral Sclerosis (ALS), Parkinson’s disease (PD) and Multiple Sclerosis (MS) stratified according to whether the neurological disease was the underlying cause of death (**a**) or a comorbid condition (**b**)

An analysis of place of death among the four neurological disease groups in which the respective disease was the underlying cause of death showed the following results (Fig. 1a): hospital was the most common place of death for patients with brain tumours, PD and MS, while private home was the leading place of death for patients with amyotrophic lateral sclerosis (ALS), with hospital ranking a close second. Private home was the second most common place of death in both brain tumours and MS, whereas nursing homes ranked second in PD. Approximately one fifth of people with brain tumours died in hospice, compared with only a minority of ALS patients, and very few patients with MS or PD. Differences in place of death between patients in whom the neurological disease was the underlying cause of death (Fig. 1a) and those in whom it was recorded as a comorbidity (Fig. 1b) were statistically significant only for Parkinson’s disease and brain tumours. To ensure a consistent analytical dataset across comparisons, cases in which a neurological diagnosis was the underlying cause of death but a concomitant neurological disease was also present were excluded from this calculation.

**Fig. 2:**
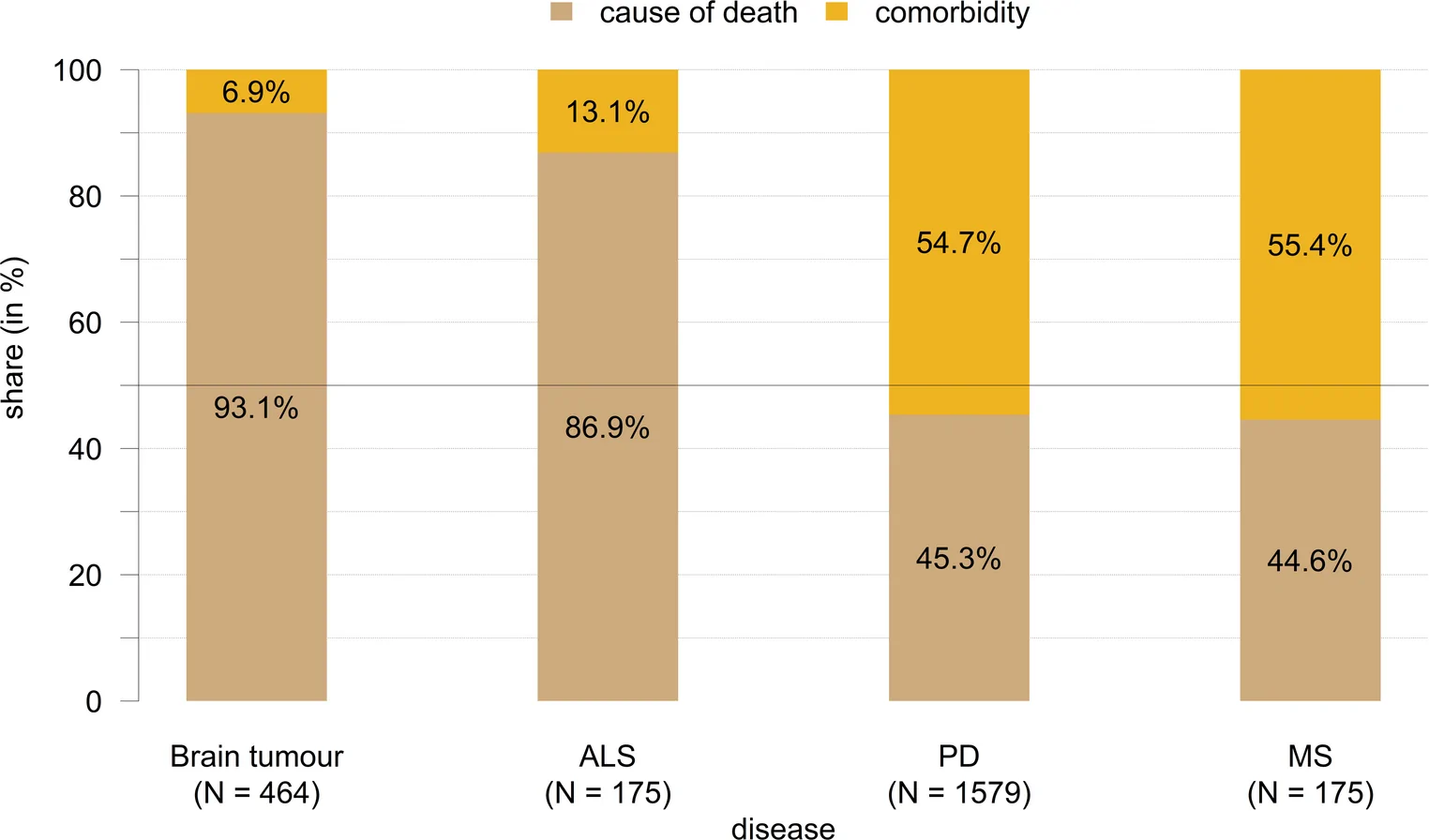
Role of neurological disease as documented on the death certificate as either cause of death or comorbidity (brain tumours, Amyotrophic Lateral sclerosis/ALS, and Parkinson’s Disease/PD, Multiple Sclerosis/MS)

According to the death certificates, the vast majority of people with brain tumours (93%) and ALS (87%) died as a direct consequence of their underlying disease. In contrast, PD and MS were reported as comorbidities in 55% of cases, meaning that they were present but not considered causally related to death on the death certificate (see Fig. 2), as classified by the physician performing the external post-mortem examination.

Notably, cases of conventional or assisted suicide (*n* = 23) were classified as “neurological disease as a comorbidity”, since the neurological condition was not the immediate cause of death. This aspect is particularly relevant for ALS (*n* = 23 with ALS as a comorbidity/Fig. 1b), which includes 6 cases of assisted or conventional suicide, and for brain tumours (*n* = 32 as a comorbidity/Fig. 1b), including 4 suicide cases, and should be taken into account when interpreting the data.

## Discussion

This study provides a population-based analysis of place and cause of death among patients with chronic neurological diseases in Munich, a large metropolitan area with a particularly dense healthcare infrastructure. Using all death certificates issued over a four-year period, this study represents a complete population-based assessment of mortality among individuals with chronic neurological diseases in the city of Munich. Overall, 4.2% of all deceased individuals had at least one chronic neurological condition.

Hospital was the most common place of death, with half of these deaths occurring in either intensive/intermediate care or in palliative care units. Nearly half of all patients died at home or in nursing homes, whereas hospice deaths remained rare (Table 2). The low proportion of hospice deaths may reflect structural limitations of the local care system rather than patient preferences alone. We previously demonstrated that patients with neurological diseases are markedly underrepresented in inpatient hospices in Germany despite their substantial contribution to population mortality [9], suggesting persistent barriers to hospice access. Consequently, the comparatively high proportion of home deaths observed in our cohort may reflect not only patient preferences but also the limited availability of suitable alternative care settings.

In 57.3% of cases, the neurological disease was regarded as causally leading to death. However, the dichotomous classification of neurological diseases as either causally leading to death or as a comorbidity may not fully capture their clinical relevance. This limitation becomes particularly evident in cases of conventional or assisted suicide, which are systematically classified as “neurological disease as a comorbidity”, as the neurological condition itself is not recorded as causative of death. As a result, the contribution of neurological diseases to death may be underestimated. This effect is particularly relevant for ALS with 6 of 23 (26%) cases listed as comorbidity having died of assisted or conventional suicide. Importantly, official mortality statistics, such as those provided by the German Federal Statistical Office, are based on this classification system, and therefore reflect this structural limitation.

Respiratory failure and related complications represented the most frequent immediate causes of death, occurring in approximately one quarter of the total cohort (Table 3). The study period overlapped with the COVID-19 pandemic and COVID-19 was recorded as the immediate or underlying cause of death in 5% of cases, a proportion comparable to that reported in the general population of Munich [10]. Importantly, patients who died from COVID-19 showed a similar distribution of place of death as the overall cohort, with hospitals and nursing homes remaining the predominant settings. Likewise, the overall distribution of place of death remained largely unchanged compared with pre-pandemic patterns [11].

Subgroup analysis based on the neurological diagnosis revealed marked differences in the place of death (Fig. 1a and b). Consistent with previous studies and patient preferences [12], patients with ALS most frequently died at home, although hospital deaths remained common. This pattern differs across healthcare systems internationally, with hospital death rates of approximately 47% reported in Canada [13], whereas integration of specialist palliative care in Sweden has been associated with substantially lower hospital death rates [14]. PD patients, in contrast, predominantly died in hospitals or nursing homes, with hospice utilization remaining extremely low, which is similar to findings from the United Kingdom [15, 16] and Canada [17]. PD is often not recorded as the immediate or underlying cause of death [16], underscoring the challenge of recognizing PD as a terminal condition. However, evidence suggests that advance care planning in PD significantly increases the likelihood of dying in the preferred place, typically at home [18]. Patients with brain tumours showed the highest rate of hospice utilisation but also a high proportion of hospital deaths. This likely reflects rapid clinical deterioration, late referral to palliative care services [19, 20], high symptom burden and difficulty in accurate prognostication [21]. Multiple sclerosis (MS) patients showed mixed outcomes: while a relevant proportion died at home, hospital deaths remained most frequent. Acute infections and complications in advanced disease likely exceed the capacity of home-based care, indicating a need for strengthened interdisciplinary community care [22]. The observed heterogeneity in place of death across diagnostic groups highlights important opportunities to improve goal-concordant care. High rates of hospital death in most conditions indicate ongoing challenges in managing complex neurological symptoms outside acute care settings.

To improve goal-concordant care, early integration of non-disease-specific palliative screening in neurological care, particularly for PD and MS, should be considered. This may support timely advance care planning and earlier referral to community-based palliative services, which has been shown to increase the likelihood of home death [17]. In addition, expanded specialist palliative care consultation services and structured implementation of advance care planning in nursing homes may increase care consistency with care preferences, reduces unwanted hospital admissions and improve end-of-life care quality [23].

A substantial proportion of death certificates documented non-specific terminal physiological mechanisms such as cardiorespiratory failure (Table 3). This highlights inherent limitations in death certification practice, as physicians are often unable to reconstruct the full causal pathway between the underlying neurological disease and terminal complications [24]. This uncertainty is further compounded by the limited availability of clinical autopsies. When performed, autopsies in our cohort were predominantly forensic with a focus on excluding external causes of death rather than elucidating disease progression. Consequently, the absence of systematic clinical autopsy practice limits the validation of death certificate data. In Bavaria, findings from completed autopsy reports are routinely transmitted to the State Office for Statistics and may lead to revision of the officially recorded cause of death when discrepancies are identified. While autopsies can therefore contribute to the validation of cause-of-death data, other measures include appropriate training of physicians and systematic reviews of death certificates by public health authorities to improve completeness and plausibility of death certificates and may contribute to more accurate cause-of-death statistics [25].

Despite these limitations, death certificate data remain a valuable population-based source for examining end-of-life circumstances in this patient population and therefore provide important, albeit proxy, insights into care patterns. Notably, while the recorded cause of death is subject to uncertainty, the place of death can be considered a robust and consistently documented variable.

### Limitations

This study is subject to several limitations inherent to the use of mandatory death certificates. The analysis is based on routinely collected administrative records, which depend on the accuracy and completeness of physician documentation. Variability in how physicians assign underlying and immediate causes of death may have introduced misclassification bias, particularly for chronic neurological diseases such as Parkinson’s disease and multiple sclerosis, which may be underreported as primary causes of death. As death certificates do not in all cases record the specialty or level of qualification of the certifying physician, we could not assess whether the physician’s specialization influenced the attribution of the cause of death, and a potential specialty-related classification bias cannot be excluded. This especially applies to deaths occurring in hospital. The dichotomous classification of diseases as either cause of death or comorbidity may lead to systematic underestimation of the contribution of chronic neurological diseases in specific contexts, such as cases of assisted or conventional suicide. The use of non-specific terminal categories such as cardiorespiratory failure reduces diagnostic precision and limits the identification of the underlying cause of death. The dataset does not include information on access to palliative care services, which may have influenced both place and cause of death. Furthermore, the study is based on data from Munich, a metropolitan area with a particularly high level of healthcare provision. Therefore, the findings may not be fully generalisable to rural regions or areas with less developed specialist and palliative care infrastructure.

## Conclusion

This population-based analysis using death certificates demonstrates substantial heterogeneity in the place and recorded cause of death among people with chronic neurological diseases in Munich, Germany. The high proportion of hospital deaths highlights opportunities for earlier advance care planning and more goal-concordant end-of-life care. Limitations in the attribution of neurological diseases as causes of death also highlight the need for improved death certification and cautious interpretation of cause-of-death statistics. The population-based data on place of death may serve as a useful reference for evaluating and comparing palliative care interventions and models of care for people with chronic neurological diseases.

## Data Availability

No datasets were generated or analysed during the current study.
